# Effects of Cholesterol in Stress-Related Neuronal Death—A Statistical Analysis Perspective

**DOI:** 10.3390/ijms21082905

**Published:** 2020-04-21

**Authors:** Maher A. Dayeh, George Livadiotis, Farzan Aminian, Kwan H. Cheng, James L. Roberts, Nikita Viswasam, Saber Elaydi

**Affiliations:** 1 Space Science and Engineering Division, Southwest Research Institute, San Antonio, TX 78238, USA; glivadiotis@swri.edu; 2 Department of Physics and Astronomy, University of Texas at San Antonio, San Antonio, TX 78249, USA; 3 Neuroscience Program, Departments of Biology, Mathematics, Engineering and Physics & Astronomy, Trinity University, San Antonio, TX 78212, USA; faminian@trinity.edu (F.A.); kcheng1@trinity.edu (K.H.C.); james.roberts@trinity.edu (J.L.R.); nviswasa@gmail.com (N.V.); selaydi@trinity.edu (S.E.)

**Keywords:** dementia, Alzheimer’s disease, oxidative stress, cellular viability

## Abstract

The association between plasma cholesterol levels and the development of dementia continues to be an important topic of discussion in the scientific community, while the results in the literature vary significantly. We study the effect of reducing oxidized neuronal cholesterol on the lipid raft structure of plasma membrane. The levels of plasma membrane cholesterol were reduced by treating the intact cells with methyl-ß-cyclodextrin (MßCD). The relationship between the cell viability with varying levels of MßCD was then examined. The viability curves are well described by a modified form of the empirical Gompertz law of mortality. A detailed statistical analysis is performed on the fitting results, showing that increasing MßCD concentration has a minor, rather than significant, effect on the cellular viability. In particular, the dependence of viability on MßCD concentration was found to be characterized by a ~25% increase per 1 μM of MßCD concentration.

## 1. Introduction

The connection between plasma cholesterol levels and the development of dementia is still an inconclusive topic of discussion in the scientific community. Results in the literature range from no effect, positive effect, and negative effect of the plasma cholesterol levels on the progress of dementia. Research has shown that the neuronal plasma membrane does more than just retaining an electrochemical gradient between the intracellular and extracellular environment. It maintains the appropriate level of fluidity for proper movement of integral membrane proteins. Research has shown that the actual lipid composition in a particular environment can significantly affect the functioning of these integral membrane proteins. The clearest example of this is the role of cholesterol and sphingolipid enriched micro domains in the plasma membrane, referred to as detergent resistant “lipid rafts” (e.g., see [1]). These rafts have reduced fluidity within their domain; yet, many receptors, enzymes and integrin proteins are found to have direct associations with these lipid microdomains [2]. It is also known that removing plasma membrane cholesterol using the biochemical agent methyl-ß-cyclodextrin (MßCD) cause the lipid raft domains to dissolve, presumably because the level of cholesterol necessary to preserve their integrity is not maintained. This disassociates the receptors/enzymes/integrins associated with them, which could compromise their integrated signaling capability [3]. For details on how cholesterol carriers may support lipid transport and injury repair in the brain and the main genetic determinants of Alzheimer disease (AD), see: [4], [5]. Once membrane lipids have been exposed to free radicals, a process of autoperoxidation may be initiated that may, if uninterrupted, proceed for years. Free radicals are highly reactive molecules with at least one unpaired electron in their outermost shell. Any free radical involving oxygen, referred to as reactive oxygen species (ROS), is a common outcome of normal aerobic cellular metabolism ([6], [7]). The built-in antioxidant system of the body plays a decisive role in the prevention of any loss due to ROS overproduction. The imbalance between the production of ROS and the biological system’s ability to detoxify the reactive intermediates leads to the oxidative stress [8]. Such stress has been implicated in the progression of major neurodegenerative diseases, namely, the AD and Parkinson’s disease [6].

In general, oxidative stress refers to the shift in the balance between oxidants and antioxidants in favor of oxidants. Regulation of the reducing and oxidizing (redox) state is critical for cell viability, activation, proliferation, and organ function. Therefore, oxidative stress in cell biology refers to the inability of a cell or organism to handle ROS, either due to increased production of ROS or due to decreased antioxidant defense enzymes. Aerobic organisms have integrated antioxidant systems, which include enzymatic and nonenzymatic antioxidants, that are usually effective in blocking harmful effects of ROS. For instance, Glutathione and antioxidant enzymes have complementary roles [9]. However, it should be noted that in pathological conditions, the antioxidant systems may be overwhelmed and sometimes ignored (for example, see: [10]).

Several studies considered that the oxidative stress and H_2_O_2_ production in neurons occur with the slow accumulation of minor damaged proteins or oxidized protein and lipid, that does not get repaired until such accumulation leads to a serious interruption of neuronal function, such that the cell undergoes apoptosis [11,12,13]; however, this may not be necessarily correct, as oxidative stress occurs as a result of either decreasing antioxidant defense systems or increasing ROS production. Cholesterol is also oxidized in the brain and results in cholesterol turnover from the membrane and replacement with fresh cholesterol to maintain appropriate levels of cholesterol for proper membrane function. We hypothesize that repeated or long-term stress and/or loss of the replacement of cholesterol will not result in reduced plasma membrane levels of cholesterol. The latter is known to affect lipid raft structure and other aspects of plasma membrane function, making the neuron more susceptible to oxidative stress.

In addition, and as noted by [14], most studies suggest that an increased content of cholesterol in the brain correlates with an increased risk of developing AD. However, not all studies support this view, and epidemiological studies on the association between plasma cholesterol levels and the development of dementia have produced conflicting conclusions (e.g., see [15,16,17,18,19,20,21]). Nevertheless, it has to be stressed that in this work we only report the effect of MßCD in the model of H_2_O_2_.

Several cultured cell models of neurons exist for dopaminergic neurons. The most direct system for studying the effects of toxins on midbrain dopaminergic neurons is the N27 neuronal cell created from fetal embryonic midbrain neurons by transformation with SV40 large T antigen [22]. This neuronal cell line has been utilized to study H_2_O_2_ toxicity [23], which we use in this work to test our hypothesis of the deleterious effects of reduced cholesterol on the neuronal response to oxidative stress.

The main scope of this study is to examine the effect of reducing oxidized neuronal cholesterol on the lipid raft structure of plasma membrane. Results reveal that there is a minor effect of altering neuronal cholesterol on cell toxicity by adding hydrogen peroxide based on 6 triplet experiments. We present the experimental results and the interpretations, and indicate a list of limitations pertaining to this project.

## 2. Materials and Methods

### 2.1. Culture of Neurons/N27 Cells

The rat immortalized mesencephalic dopaminergic neuronal cell line 1RB3AN27 (N27) was used (each vial contains ≥ 1 × 10^6^ viable cell; see also [22]). The cell line was a kind gift from Dr. Curt Freed (University of Colorado Health Science Center, Denver CO). Cells were cultured at 5% CO_2_ at 37°C in RPMI 1640 medium (Mediatech, Inc., Manassas, VA, USA), supplemented with 10% fetal bovine serum (FBS) (Mediatech, Inc.), 1% 10,000 μg/mL penicillin streptomycin (Mediatech, Inc), and 1% 200 mM L-alanyl-L-glutamine (ATCC, Manassas, VA, USA) and subcultured weekly. All experiments were performed with cells plated in 12- well Cell Culture Multiwell plates (Greiner Bio One, Munich, Germany), at approximately 100,000 cells/well; (24 h each experiment, all performed in a week). This cell line has previously been used as a model to study neurotoxicity on dopamine neurons (e.g., [23,24,25,26]). The sample size is *n* = 8; note that 24 wells (in 2 plates) have been used in performing each experiment, which were grouped in 8 independent triplets of samples of the same concentration [27]. To account for variability or error in the cell-counting readings, we have a blank plate that is read by the machine before each experiment that served as a background level, while we performed triplet experimental trials.

### 2.2. Calcein AM Viability Assay

**Figure 1:**
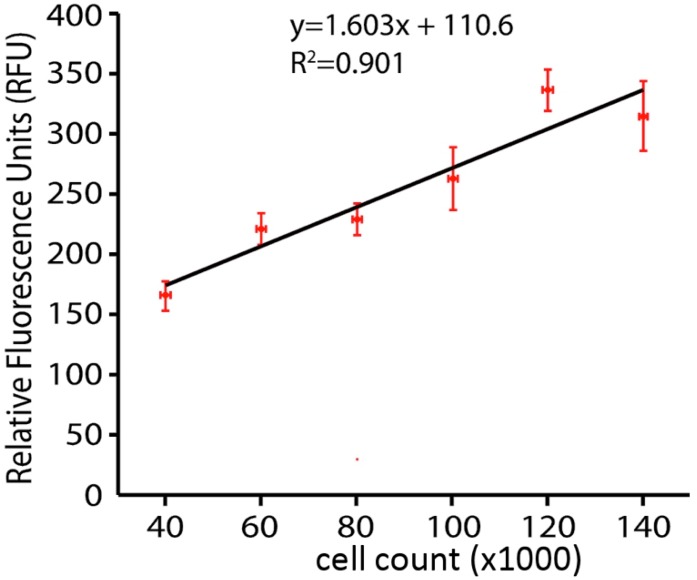
Linear relationship between cell number and calcein fluorescence.

A calcein AM viability assay was conducted. Cells were washed with Hank’s Balanced Salt Solution (HBSS) (Mediatech, Inc) and incubated with HBSS containing 1 μg/mL Calcein AM (Life Technologies, Eugene, Oregon, USA) in DMSO and 20% Pluronic F-127 (Life Technologies) in darkness for 20 min at 37°C. After incubation cells were washed again with HBSS and fluorescence read in a SpectraMax M4 Microplate Reader at excitation wavelength 495 nm and emission wavelength 515 nm, the validity of 6assay method was tested for determining the number of live cells by a whole plate fluorescence assay by using the Calcein AM live cells reagent on culture plates inoculated with known amounts (serial dilution) of N27 cells. Data plotted in Figure 1 show a linear relationship up to the 100,000 cells per well, which is maximal in our subsequent hydrogen peroxide toxicity studies. We thus interpret our fluorescent analysis to mean that if hydrogen peroxide reduces the amount of fluorescence in the dish by half, then half of the cells have been killed and are no longer capable of taking up and concentrating the Calcein AM dye.

### 2.3. Neuron Death Behavior

**Figure 2:**
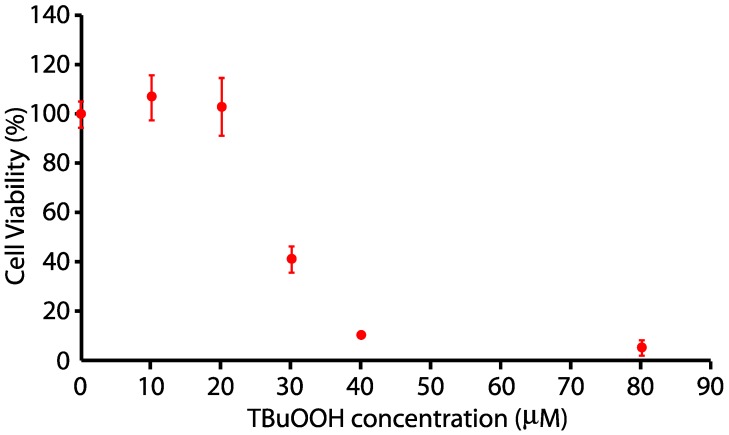
Dose-response toxicity curve for tert-butyl hydrogen peroxide on N27 neuronal cells.

N27 cells were plated at 100,000 cells/well in RPMI 1640 as indicated above. At the appropriate density, cells were changed to 0% FBS for 24 h, and cells were exposed to 0, 10, 15, 20, 25, 30, 40, and 80 µM tert-Butyl hydrogen peroxide (tBuOOH) for 24 h. N27 cells were killed in a dose-dependent fashion after 24 h of treatment with tert-butyl H_2_O_2_, with a half-maximal dosage at approximately 24 µM, as indicated in Figure 2.

### 2.4. Cholesterol Assay and MßCD-Cholesterol Modeling

Cells were washed with HBSS and dissolved in 0.1% PBS-Triton x 100, transferred to eppendorf tubes and spun at 4000 rpm for 5 min. The Amplex Red assay protocol (Life Technologies) was performed with the following modifications. Samples were prepared in 1x Reaction Buffer in a 96 well microplate, exposed to a working solution of 300 μM Amplex Red reagent stock solution (Amplex Red reagent, horseradish peroxidase, cholesterol oxidase, cholesterol esterase) and incubated for 30 min at 37 °C. A cholesterol standard curve was prepared using a cholesterol reference standard in 1× Reaction Buffer, to yield samples with cholesterol concentrations between 0 and 8 μg/mL. Fluorescence was measured in a SpectraMax M4 Microplate Reader at excitation wavelength 535 nm and emission wavelength 590 nm. The averaged values of MßCD (in [μM]) and normalized cholesterol content are shown in Table 1.

### 2.5. MßCD-tBuOOH Cell Viability Assay and Modeling

N27 cells were plated in the earlier stated format. After suspension in 0% FBS RPMI for 24 h, cells were exposed to 0–2.5 μM MßCD for 1 h. Cells were then washed with HBSS and exposed to previously stated concentrations of tBuOOH for 24 h. A calcein AM viability assay was conducted as described above.

Table 2 shows the triplet cellular viability experiments for different H_2_O_2_ doses and MßCD concentrations. Inferred cellular viability at different H_2_O_2_ dosage and for different MßCD concentrations is shown in Table 3. Note that in general, the cell viability of the nanoparticle-treated samples is less than the control sample (100%), but it is also possible that for few treatments, there might be values that exceed >100%, that is, the value is higher than the standard value. The reason for the latter case is that in order to calculate viability, we need to know the optical density value of a non-treated/standard/control population of cells (that is, the theoretical 100%). This may mean that a low treatment can result in an optical density value that is higher than the 100%. Also, some treatments may help cells proliferate, giving you more than 100%, for instance, see: [28].

## 3. Results

### 3.1. Methyl-ß-Cyclodextrin (MßCD) Depletion of Plasma Membrane Cholesterol

**Figure 3:**
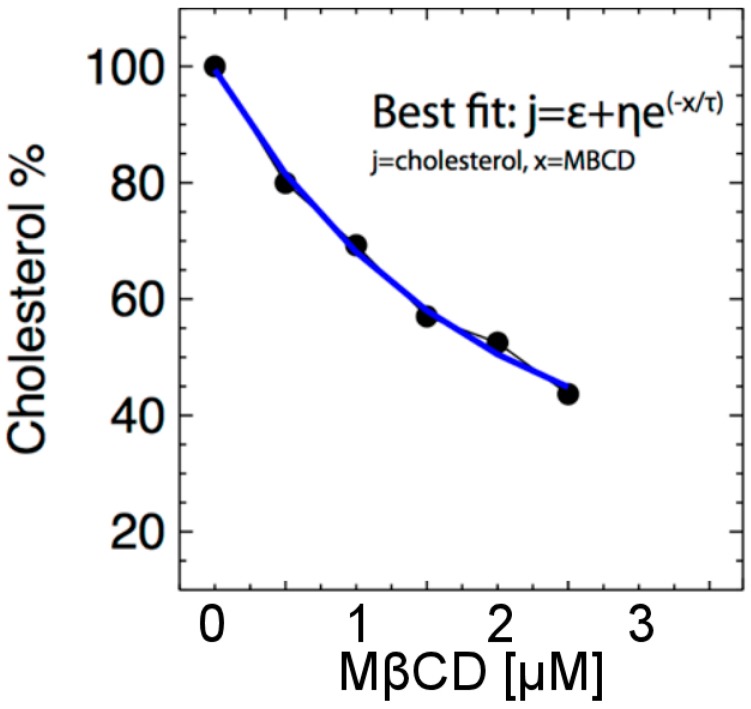
Cholesterol concentration (denoted by j) is depicted as a function of the concentration of MßCD. As MßCD increases, the cholesterol level decreases exponentially.

The levels of plasma membrane cholesterol can be reduced by treating the intact cells with MßCD (0–10 µM), a cyclic molecule with a structure that extracts a cholesterol molecule from the surface of the cell (plasma membrane). However, 5 µM or more of MßCD, while extracting more cholesterol from the cell, also caused ~50% cell death (data not shown), so in this paper, we focused on 0–2.5 µM MßCD. Using data from Table 1, we examine the cholesterol (%) variability as a function of MßCD (μM) content. As shown in Figure 3, MßCD caused a dose-dependent reduction in the level of total cholesterol in the N27 neuronal cell. We then fit the relationship between MßCD and cholesterol by the equation:

(1) $$j(x)=\varepsilon +\eta \;e^{-x/\tau }$$

where *j* is the cholesterol % concentration and *x* represents MßCD concentration. The function *j*(*x*) decreases exponentially with increasing *x*, where *ε* is its lower asymptote, *τ* is the difference between its upper and lower asymptotes, and *η* is a regulating parameter. Note: This clearly monotonically decreasing relationship between MßCD and cholesterol will be used to analyze the detected relationship between H_2_O_2_ and MßCD and derive the converted relationship between H_2_O_2_ and cholesterol.

### 3.2. Effect of MßCD’s reduction of Plasma Membrane Cholesterol on the Sensitivity of N27 Cells

**Figure 4:**
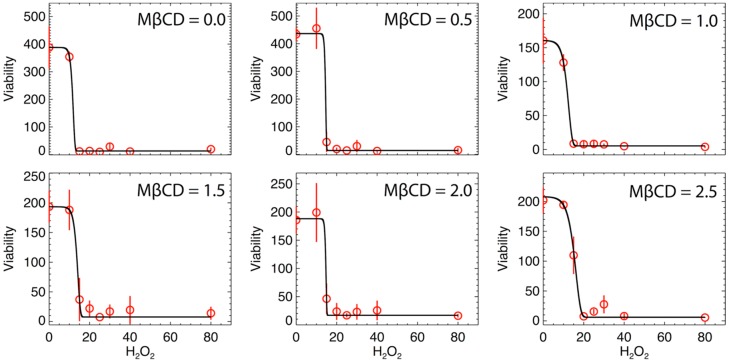
Viability as a function of H_2_O_2_ (µM) for different values of MßCD concentrations (µM). The solid black curve is the best fit of the model (see text), and the red circles are the averaged values of the triplet viability measurements at each H_2_O_2_ concentration. Error bars represent the standard deviations of the triplet measurements.

We hypothesized that reducing plasma membrane cholesterol and the subsequent disruption of lipid raft entities in the plasma membrane would not increase the sensitivity of N27 cells to oxidative stress and thus, resulting in a leftward shift in the H_2_O_2_ dose response curve. Figure 4 shows the variation of cellular viability as a function of H_2_O_2_ (µM) for different values of MßCD concentrations. Here, data were fit by a modified version of Gompertz law of mortality, which was often used in previous studies for describing the cellular viability and fitting data that involve growing populations with an inflection point. For instance, see: [29,30]. This model has never been used in this specific case of viability with H_2_O_2_. The modeling provides a good fit. The expression of the modelled cell viability is:

(2) $$v(h)=\alpha +\beta \;\exp[-e^{\gamma (h-\delta )}]$$

where *v* is the cell viability, *h* is the concentration level of H_2_O_2_, *α* is the asymptote as *h* becomes large, *γ* represents the effect of MßCD (if any) on the toxicity of the neuronal cell, *β* is proportional to the difference between the upper and lower asymptotes, and *δ* represents the toxic intensity of H_2_O_2_.

On each panel of Figure 4, the error bars are inferred from the variance of the triplet mean at each H_2_O_2_ value and represent the variability of the triplet data. The mean and variance are trivially given by a sampling of three measurements, i.e., $\bar{v}=(1/3)\cdot \sum_{i=1}^{3}v_{i}$, $\sigma_{v}^{2}=(1/2)\cdot \sum_{i=1}^{3}{\left(v_{i}-\bar{v}\right)}^{2}$. Then, the fits in Figure 4 were optimized by minimizing the chi-square, determined by the differences between *n* = 8 pairs of observed ${\bar{v}}_{m}$ and modeled $v(h_{m};\alpha ,\beta ,\gamma ,\delta )$ values of the viability, that is, $\chi_{}^{2}=(1/4)\cdot \sum_{m=1}^{8}\left\{\sigma_{v_{m}}^{-2}\cdot {\left[{\bar{v}}_{m}-v(h_{m};\alpha ,\beta ,\gamma ,\delta )\right]}^{2}\right\}$ (where we have 8 data points, but 4 modeled parameters to estimate their optimal values). Table 4 lists the values of the optimized parameters inferred from the best-nonlinear fit for all MßCD concentrations.

**Figure 5:**
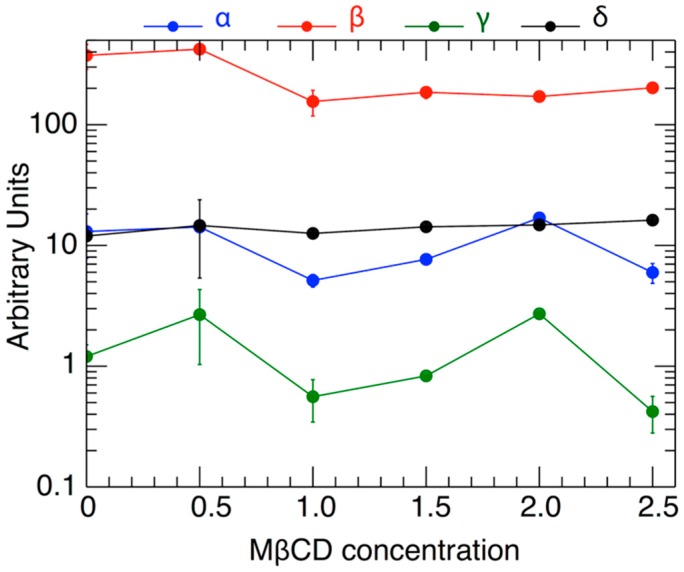
The values of the parameters *α*, *β*, *γ*, *δ*, and their standard deviations, as shown in Table 4, plotted against the respective values of the MßCD concentration (the latter’s standard deviations are not plotted).

**Figure 6:**
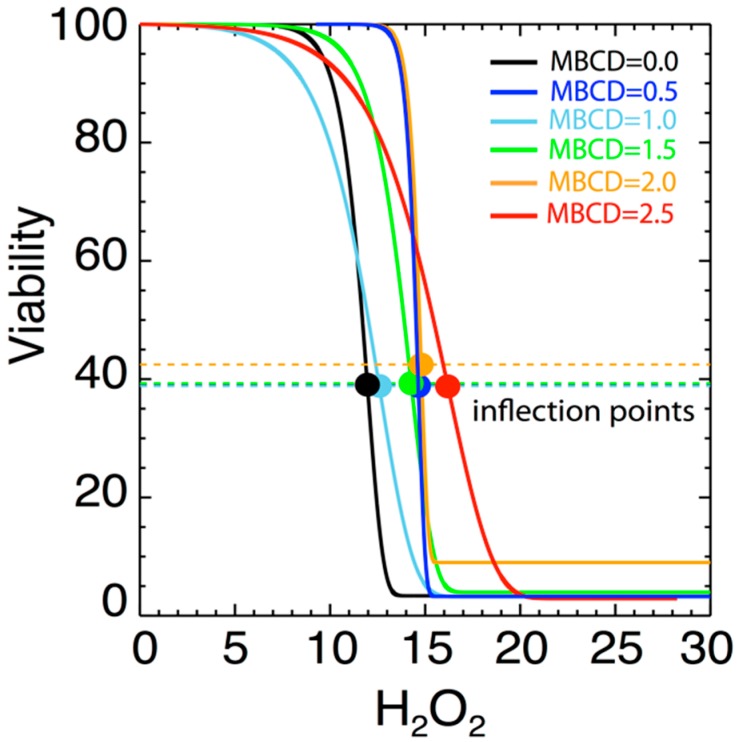
Diagrams of fitted curves in Figure 4, normalized to superposed viability curves for different MßCD concentrations. A small variation of the location of the inflection point *δ* with the MßCD concentrations is observed.

Figure 5 shows the variation of the fitted parameters as a function of H_2_O_2_ level and as a function of varying MßCD concentration. Two parameters of physical importance are *γ* (shown in black) and represents the effect of MßCD on the toxicity of the neuronal cell), and *δ* (shown in green) and represents the toxic intensity of H_2_O_2_. Figure 6 shows the normalized fits from all the 6 triplet experiments on the same plot and color-coded to indicate different MßCD concentrations. In particular, we over-plotted the inflection points of each of the fitted curves (denoted by solid circles). The inflection point is a good measure of the gradual viability drop as H_2_O_2_ increases. As can be determined from Equation (2), the inflection point is located at an H_2_O_2_ value of *h = δ*, and corresponds to the viability value

(3) $$\nu_{\inf}=\alpha +e^{-1}\beta$$

As shown, the inflection points are characterized by some variability at different concentrations. There is a weak dependence of the inflection point’s location *δ* on MßCD concentrations. This indicates that there is a small effect of cholesterol on the viability. The statistical confidence of this variability is estimated in the next section.

## 4. Statistical Analysis

We examine the set of the estimated parameter values, *p* = *α*, *β*, *γ*, *δ*, to assess whether they are independent of the MßCD concentration or not. The following statistical analysis samples the values derived in Section 3 and shown in Table 4; thus, there are *n* = 6 estimates for each parameter value.

For the hypothesis of the parameters being constant and independent of the MßCD concentration, we use the constant statistical model (set as *p*) and derive the value of the corresponding minimized chi-square and its reduced value. Indeed, for any *p* = *α*, *β*, *γ*, *δ*, we have

(4) $$\chi_{p}^{2}(p)=\sum_{i=1}^{6}\left[\sigma_{p}^{-2}{}_{i}\cdot {\left(p_{i}-p\right)}^{2}\right];\;\chi_{p,\;}^{2}{}_{\mathrm{red}}=(1/5)\cdot \chi_{p}^{2}$$

whose minimization gives

(5a) $$\chi_{p}^{2}{}_{\min}=\sum_{i=1}^{6}\left[\sigma_{p}^{-2}{}_{i}\cdot {\left(p_{i}-\bar{p}\right)}^{2}\right];\;\bar{p}=\frac{\sum_{i=1}^{6}\sigma_{p_{i}}^{-2}p_{i}}{\sum_{i=1}^{6}\sigma_{p_{i}}^{-2}}$$

and

(5b) $$\delta p=\sqrt{\frac{(1/6)\cdot \sum_{i=1}^{6}\sigma_{p}^{-2}{}_{i}}{{\left(\sum_{i=1}^{6}\sigma_{p}^{-2}{}_{i}\right)}^{2}-\sum_{i=1}^{6}\sigma_{p}^{-4}{}_{i}}\cdot \chi_{p}^{2}{}_{\min}+\frac{1}{\sum_{i=1}^{6}\sigma_{p}^{-2}{}_{i}}}$$

(Note: the error of the mean is given as the square sum of two different types of errors, the statistical and propagation errors. The former is related to the chi-square minimization, is caused by the variability of the sampled values, and it has geometric interpretation; the latter is caused by the propagation of the errors of the sampling values. For more applications on these two types of errors, see: [31,32,33,34].)

To test the hypothesis of the parameters dependence on the MßCD concentration, we use the linear model, set as $p=a_{p}+b_{p}\cdot Mß\mathrm{CD}$, where the two model parameters *a_p_* and *b_p_* correspond to the intercept and slope. The chi-square is then set as

(6) $$\chi_{p}^{2}(a_{p},b_{p})=\sum_{i=1}^{6}\frac{{\left[p_{i}-(a_{p}+b_{p}\cdot MßCD_{i})\right]}^{\;2}}{\sigma_{p}^{2}{}_{i}+b_{p}^{2}\cdot \sigma_{MßCD\;}^{2}{}_{i}};\;\chi_{p,\;}^{2}{}_{\mathrm{red}}=\frac{1}{4}\chi_{p}^{2}{}_{\min},\;\mathrm{for}\;p:\;\alpha ,\;\beta ,\;\gamma ,\;\delta$$

where $\sigma_{MßCD\;}^{2}$ are the uncertainties on the values of MßCD concentrations. Note that the minimization of chi-square as shown in Equation (6) leads to two complicated normal equations that can only be solved numerically; (for more details, e.g., see: [35]). The same applies for the equations describing the errors of the intercept and the slope (see: [31,35]).

There are two easily derived and statistically confident tools for estimating the goodness of the fitting. The reduced chi-square and the P-value; both are derived from the estimated minimum value of chi-square $\chi_{\min}^{2}$.

The reduced chi-square $\chi_{\mathrm{red}}^{2}$ helps to estimate the goodness of the fitting, and is generally given by $\chi_{\mathrm{red}}^{2}=(1/n)\cdot \chi_{\min}^{2}$, where *n = Ν−M* represents the degrees of freedom, while *N* is the number of samples and *M* is the number of free parameters to be estimated by the fitting. The goodness of fitting is characterized as better when the estimated value of $\chi_{\mathrm{red}}^{2}$ is closer to 1; if $\chi_{\mathrm{red}}^{2}$ is smaller (greater) than 1, the involved errors are said to be overestimated (underestimated) [36].

The *p*-value is derived from the chi-square distribution. It is the portion of the distribution corresponding to all the extremer values of chi-square than the estimated one, $\chi_{\min}^{2}$. The chi-square distribution is written as

(7) $$f(x;n)=\frac{2^{-\frac{1}{2}n}}{\Gamma (\frac{1}{2}n)}\cdot x^{\frac{1}{2}n-1}\cdot \exp\left(-\frac{1}{2}x\right),$$

while the *p*-value is given by:

(8) $$P-\mathrm{value}(\chi_{\min}^{2})=\mathrm{MIN}\left[\int_{\;0}^{\chi_{\min}^{2}}f(x;n)\;dx\;\;,\;\;\int_{\;\chi_{\min}^{2}}^{\infty }f(x;n)\;dx\right].$$

The goodness of the fitting is characterized better when the estimated *p*-value is larger and closer to 0.5. Hypotheses, corresponding to *p*-values larger than ~0.05 confidence, are considered acceptable. The behavior of both measures are similar when they are standardized to be minimized on the best fitting goodness, such as, 1-2⋅P-value and $\left|\chi_{\mathrm{red}}^{2}-1\right|$. A common measure can be considered by the formula:

(9) $$\mathrm{Common}\;\mathrm{Measure}\;\equiv \sqrt{{\left(1-2\cdot P-\mathrm{value}\right)}^{2}+{\left|\chi_{\mathrm{red}}^{2}-1\right|}^{2}}.$$

For further details on the characterization of the goodness of fitting, see: [36]; for other applications of this characterization, see: [34,35,36,37,38].

**Figure 7:**
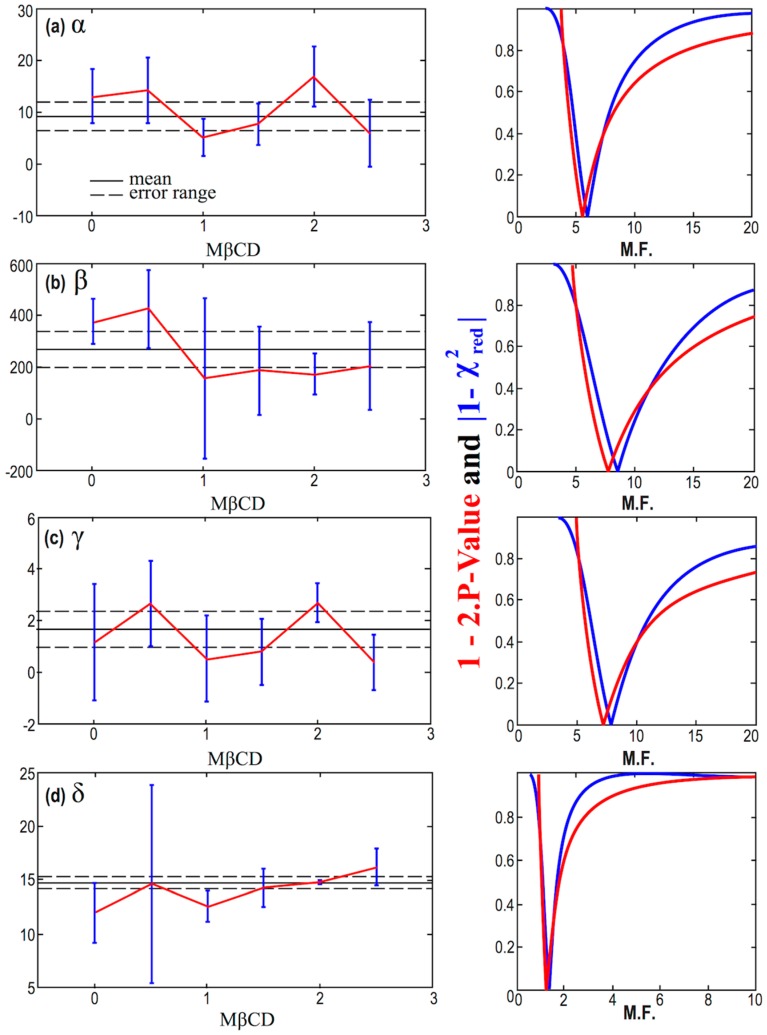
(Left column) Diagrams of the values of the parameters *α (a)*, *β (b)*, *γ (c)*, and *δ (d)*, plotted against the concentration of MßCD, after accounting for the optimal multiplication factor (M.F.). (Right column) Corresponding diagrams of the modified *p*-value and reduced chi-square characterizing fitting of the parameters *α*, *β*, *γ*, and *δ*, modeled as constants with MßCD, and plotted as a function of the multiplication factor (M.F.)

**Figure 8:**
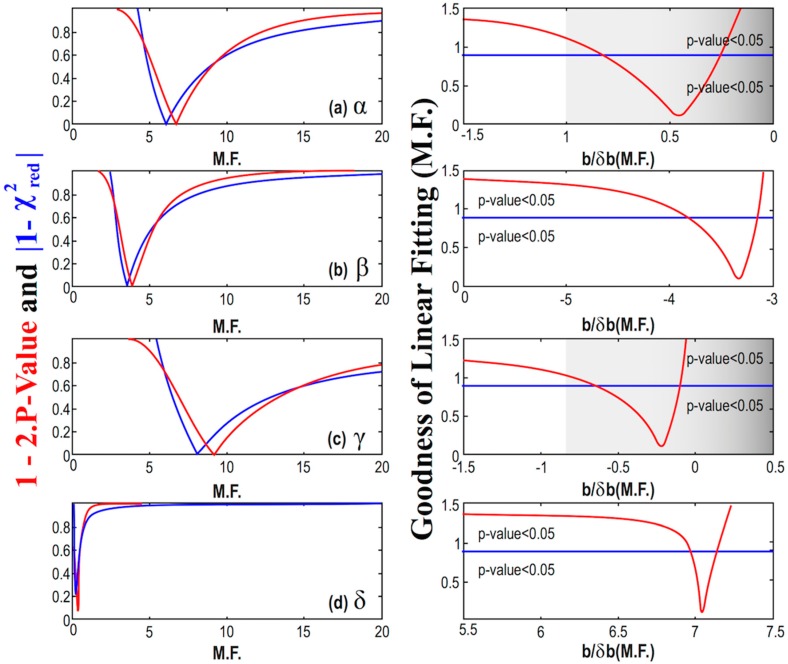
(Left) Diagrams of the modified *p*-value and reduced chi-square characterizing the linear fitting of the parameters *α (a)*, *β (b)*, *γ (c)*, and *δ (d)*, with MßCD plotted, as a function of the multiplication factor (M.F.). We identify the M.F. value, for which the corresponding *p*-value and reduced chi-square are optimized. The goodness of fit is shown using both the modified *p*-value and reduced chi-square. (Right) Here, the goodness of fit is shown using the unified measure as given by Equation (9) while it is depicted as a function of the signal-to-noise ratio of the slope *b/δb*, characterizing the linear fitting of the parameters with MßCD; (note: Both the ratio *b/δb* and the goodness of fitting are plotted as functions of the multiplication factor (M.F.)). The cases of the parameter α and γ have the optimal values of the *p*-value and reduced chi-square for │*b/δb*│< 1, so that the hypothesis of being dependent on MßCD is rejected. On the other hand, the cases of the parameter *β* and *δ* have the optimal values of the *p*-value and reduced chi-square for │*b/δb*│>> 1, thus, the hypothesis of MßCD dependence is accepted.

The errors of the parameters *α*, *β*, *γ*, and *δ*, in Table 4, as derived by the nonlinear fitting of the model given by Equation (2) are not quite precise, and they are likely suffer from possible underestimations or overestimations. In order to improve our results, we consider a multiplication factor (M.F.) for the smaller and questionable errors; then, we detect the optimal value of M.F. that minimizes 1-2⋅P-value and $\left|\chi_{\mathrm{red}}^{2}-1\right|$, thus, maximizes the goodness of the fitting. First, we apply this methodology for the constant statistical model, and the results are shown in Figure 7. This can be seen as an optimal averaging technique, where the results of the mean and its error are shown in Table 5. Next, we apply the methodology for the linear statistical model, and the results are shown in Figure 8.

We observe that the goodness of the fitting, which is calculated using the unified measure as given by Equation (9), is minimized for some values of the multiplication factor that do not always correspond to significant signal-to-noise ratio as regards the slope; in such a case, the linear model is disregarded. In particular, for the cases of the parameter *α* and *γ*, we have the optimal values of the *p*-value and reduced chi-square derived for small signal-to-noise ratios, i.e., │*b/δb*│< 1, so that the hypothesis of these parameters being dependent on MßCD is rejected. On the other hand, the cases of the parameter *β* and *δ* have the optimal values of the *p*-value and reduced chi-square for │*b/δb*│>> 1, thus, the hypothesis of these parameters being dependent on MßCD is accepted.

Our analysis concluded that parameters *α* and *γ* are likely constants, while parameters *β* and *δ* are likely exhibiting small linear dependence on MßCD, correlated and anti-correlated, respectively. More specifically, we obtained:

(10) $$\begin{array}{l} \alpha =9.2\pm 2.8\;\;,\\ \beta =(401\pm 57)-(109\pm 33)\cdot Mß\mathrm{CD}\;\;,\\ \gamma =1.69\pm 0.70\;\;,\\ \delta =(11.14\pm 0.48)+(1.87\pm 0.27)\cdot Mß\mathrm{CD}\;\;. \end{array}$$

All results of the performed statistical analysis are included in Table 5, where we have separated the cases of constant and linear statistical models. Furthermore, using the estimated values in Equation (10), we can derive the linearized dependence of viability on MßCD, namely,

(6) v≅(156.72±21.16)⋅[1+(0.25±0.08)⋅MßCD].

Therefore, the dependence of viability on MßCD concentration is ~25% increase per 1 μM of MßCD.

## 5. Experimental Limitations

We list below a number of important limitations characterizing the experiments and considerations involved in this study. Albeit, the next level of work is set to be performed in a future follow-up project, we find it necessary to explicitly state these limitations:

The results identified by this study are not generalizable and only report the effect of MßCD in the model of H_2_O_2_ in this particular cell line.

H_2_O_2_ represents only a model for increasing ROS production; with aging, antioxidant defense systems may decrease and therefore, a model where intracellular defense enzymes are decreased should be assessed, such as glutathione depletion/erastin treatment.

A positive control for the model must be used, since the lack of effect may not be attributed to MßCD, but simply because H_2_O_2_ does not reduce cholesterol.

Propidium iodide may be used to ensure measurements of cell viability and not proliferation.

The cell viability of nanoparticle-treated samples should ideally be kept less than the control sample (100%).

## 6. Discussion and Conclusions

A review paper by [12] indicated that several studies provided conflicting results on the association between plasma cholesterol levels and the development of dementia (e.g., [13,14,15,16]). Results range from no effect, positive effect, and negative effect on the progress of dementia.

In this study, we hypothesized that repeated or long-term stress and/or a decrease in the replacement of oxidized neuronal cholesterol would not result in reduced plasma membrane levels of cholesterol. If true, this would affect lipid raft structure and other aspects of plasma membrane function, making the neuron more susceptible to oxidative stress. Since the levels of plasma membrane cholesterol can be reduced by treatment of the intact cells with MßCD, we used MßCD (0–10 µM) in our experiment. The relationship between cholesterol and MßCD has been quantified by Equation (1).

A modified version of the Gompertz mortality function (as described in Equation (2)) was used to model the relationship between the cell viability and the level of H_2_O_2_ with varying concentrations of MßCD, and hence varying levels of cholesterol. A least-squares analysis was performed and the model parameters were inferred. Traditionally, Gompertz law of mortality is used to describe the cellular viability (e.g., [25]).

The results of the examination of the variation of viability as function of H_2_O_2_ level and the MßCD concentration showed that there is a minor rather than significant effect of altering neuronal cholesterol on cell toxicity by the addition of hydrogen peroxide, based on six triplet experiments. Cellular viability curves are similar and it is not possible from the curve-fitting analysis to identify a consistent physical trend in the fitted parameters.

In particular, the modeled parameters of physical importance are *γ*, which represents the effect of MßCD on the toxicity of the neuronal cell, and *δ*, which represents the toxic intensity of H_2_O_2_. Parameter *γ* was found to be characterized by a statistical constant model, while parameter *δ* by a linear model of the increasing MßCD concentration. Overall, the dependence of viability on MßCD concentration was found to be characterized by ~25% increase per 1 μM of MßCD concentration.

In summary, the results show that:

(1) The viability of neuron cells drops sharply to stable minimum at H_2_O_2_ concentrations of 15–20 (µM); the corresponding curves are well described by a modified form of the Gompertz law of mortality.

(2) The effect of increasing MßCD concentration on the viability is not conclusive, since a small increase of the location of the inflection point (corresponding to the drop of viability in (1)) was detected to be correlated with increasing MßCD concentration; this corresponds to H_2_O_2_ viability, characterized by a ~25% increase per 1 μM of MßCD concentration.

We note that there are likely other effects that could interfere with the membrane cholesterol and are not taken into account in this study. These may include age-dependent changes in membrane cholesterol (e.g., [39]), and the effects of different amyloid peptides and oligomers preparations, among others. Further studies in a controlled environment are necessary to better define the relationship between oxidative stress, membrane cholesterol, and cell viability. Also, recently, it has been shown that the modeling of chemical kinetics can be done either with differential or difference equations [40]; the mathematical framework of difference equations has been extensively over the last decades (e.g., see [41]), with particular applications on population dynamics in biological systems [42].

## Figures and Tables

**Table 1:** Measurements (average values) of Cholesterol for every methyl-ß-cyclodextrin (MßCD) value.

MßCD [μM]	Cholesterol [%]
0.0	100
0.5	79.97
1.0	69.25
1.5	57.02
2.0	52.50
2.5	43.66

**Table 2:** Triplet cellular viability experiments for different H_2_O_2_ doses and MßCD concentrations. “Exp. #” refers to individual trials of the triplet experiment.

	MβCD=0			MβCD=0.5			MβCD=1.0		
H_2_O_2_ [µM]	Exp. 1	Exp. 2	Exp. 3	Exp. 1	Exp. 2	Exp. 3	Exp. 1	Exp. 2	Exp. 3
0	473.02	328.33	362.08	461.07	431.04	410.68	182.08	177.97	121.52
10	368.59	361.37	332.60	541.67	416.46	408.28	134.25	113.8	135.96
15	11.62 *	13.06	10.18	44.82 *	81.23	8.41	8.40 *	9.58	7.23
20	13.13 *	12.98	13.28	13.60	23.54	21.59	4.25	14.39	4.127
25	9.92 *	9.01	10.836	16.10	12.12	14.56	5.90	14.39	4.12
30	27.25	15.04	44.33	25.51	10.05	54.34	8.68	6.10	7.39 *
40	11.23 *	12.07	10.39	12.44 *	14.31	10.58	4.64 *	5.13	4.16
80	19.08	14.45	25.26	11.94	10.32	24.08	1.88	5.42	3.65 *
	MβCD = 1.5			MβCD = 2.0			MβCD = 2.5		
H_2_O_2_ [µM]	Exp. 1	Exp. 2	Exp. 3	Exp. 1	Exp. 2	Exp. 3	Exp. 1	Exp. 2	Exp. 3
0	223.38	171.39	185.32	164.88	180.13	212.25	226.04	196.60	183.61
10	152.32	191.64	220.30	151.79	190.53	255.27	198.92	188.58	195.53
15	78.76	10.21	22.35	77.53	30.58	30.57	141.26	78.38	109.82 *
20	13.89	14.50	37.59	16.64	13.35	41.04	9.07	5.90	7.82 *
25	8.47	7.31	6.69	20.06	12.81	17.66	11.93	14.20	20.45
30	10.98	9.36	30.56	21.19	9.90	37.64	19.33	18.44	45.11
40	46.68	7.54	4.40	45.53	17.39	13.59	3.20	12.40	8.08
80	8.01	7.42	26.70	17.04	12.49	18.69	4.61	6.31	5.46 *

* Indicates the average of the other two experiments for the same MBCD value. This data point was discarded through the Q-test for being an outlier.

**Table 3:** Cellular viability at different H_2_O_2_ doses and for different MßCD concentrations *.

H_2_O_2_ [µM]	MβCD=0	MβCD=0.5	MβCD=1.0	MβCD=1.5	MβCD=2.0	MβCD=2.5
0	387.81 ± 75.70 **	434.26 ± 25.35	160.52 ± 33.84	193.36 ± 26.91	185.76 ± 24.18	202.08 ± 21.74
10	354.19 ± 54.19	455.47 ± 74.77	128.00 ± 12.33	188.09 ± 34.13	199.20 ± 52.28	194.35 ± 5.28
15	11.62 ± 1.44	44.82 ± 36.41	8.41 ± 1.17	37.11 ± 36.58	46.23 ± 27.11	109.82 ± 31.44
20	13.13 ± 0.15	19.58 ± 5.27	7.59 ± 5.89	21.99 ± 13.51	23.68 ± 15.13	7.48 ± 1.58
25	9.92 ± 1.29	14.26 ± 2.01	8.14 ± 5.49	7.49 ± 0.91	16.84 ± 3.69	15.53 ± 4.41
30	28.87 ± 8.87	29.97 ± 22.48	7.39 ± 1.29	16.97 ± 11.80	22.91 ± 13.95	27.63 ± 15.15
40	11.23 ± 1.19	12.44 ± 2.64	4.64 ± 0.68	19.54 ± 23.56	25.50 ± 17.45	7.89 ± 4.60
80	19.60 ± 5.42	15.45 ± 7.52	3.65 ± 1.77	14.04 ± 10.96	16.07 ± 3.21	5.64 ± 0.85

* Cellular viability (%) inferred at different MßCD concentrations (μM). ** Shown uncertainties represent standard deviations inferred from the triplet experiments, where errors would be estimated by dividing these values by the square root of 3.

**Table 4:** Fitted parameters and goodness of fit.

#	MßCD	*α*	*σ_α_*	δ*α*	*β*	*σ_β_*	δ*β*	*γ*	*σ_γ_*	δ*γ*	*δ*	*σ_δ_*	δ*δ*	$\chi_{red}^{2}$
1	0	13.05	5.22	1.85	374.8	86.9	30.7	1.20	0.30	0.11	11.96	2.00	0.71	3.02
2	0.5	14.22	1.08	0.38	422.2	18.3	6.5	2.67	1.64	0.58	14.64	9.27	3.28	0.52
3	1	5.13	0.62	0.22	155.5	37.3	13.2	0.56	0.22	0.08	12.58	1.02	0.36	1.18
4	1.5	7.67	0.69	0.24	185.7	20.8	7.4	0.83	1.27	0.45	14.27	1.28	0.45	0.59
5	2	16.93	1.01	0.36	171.2	9.6	3.4	2.72	0.10	0.04	14.79	0.08	0.03	0.19
6	2.5	5.97	1.11	0.39	202.2	20.5	7.2	0.42	0.14	0.05	16.20	1.22	0.43	1.85

Note: the errors of the parameters are derived from their standard deviation, $\delta p=\sigma_{p}/\sqrt{8}$.

**Table 5:** Statistical Analysis of Model Parameters vs. MßCD.

	Constant Model				Linear Model						
#	Mean	Error	M.F.	*r* (%)	Intercept *a*	Error *δa*	Slope *b*	Error *δb*	*b/δb*	M.F.	*r* (%)
*α*	9.2	2.8	5.8	−18.6	10.8	3.8	−1.29	2.8	−0.46	6.5	−32.7
*β*	266	70	8.2	−90.8	401	57	−109	33	−3.34	3.8	−85.6
*γ*	1.69	0.70	7.5	−8.0	1.95	1.36	−0.17	0.75	−0.22	8.7	−10.9
*δ*	14.78	0.53	1.3	95.6	11.14	0.48	1.87	0.27	7.04	0.36	96.2
